# Down regulation of anti-inflammatory cytokines in ileal mucosa associates with active disease in juvenile idiopathic arthritis

**DOI:** 10.1186/1546-0096-9-S1-P107

**Published:** 2011-09-14

**Authors:** Miika Arvonen, Paula Vähäsalo, Sami Turunen, Harri Salo, Outi Vaarala, Tuomo J Karttunen

**Affiliations:** 1 Departments of Paediatrics, University of Oulu, and Oulu University Hospital, Finland; 2 Pathology, University of Oulu, and Oulu University Hospital, Finland; 3 Immune Response Unit, IMBL3 of National Institute of Health and Welfare of Helsinki, Finland

## Background

Functional alterations of the intestinal immune system such as increased permeability have been described in juvenile idiopathic arthritis (JIA). Mechanisms of these changes and their role in the pathogenesis of JIA are unknown.

## Aim

To analyze gut mucosal immune regulatory and anti-inflammatory mechanisms in JIA and how they relate with the clinical course of the disease.

## Methods

We performed gastroduodenoscopy and colonoscopy in 16 children with JIA suffering from gastrointestinal (GI) symptoms. The control children (n=25) had GI-symptoms, but eventually were shown not to have any significant GI disease. We analyzed in duodenal and ileal biopsy samples the mRNA expression of IFNγ, TGFβ, IL6, IL4, FoxP3, IL17A, CTLA4, ICOS, GITR, IL10, IL18, IL12p40, IL23, TLR2, TLR4, and TLR5 with RT-PCR. We also assessed correlation between the clinical markers of JIA and the mucosal mRNA levels.

## Results

None of the mRNA expression levels (IFNγ, TGFβ, IL6, IL4, FoxP3, IL17A, CTLA4, ICOS, GITR, IL10, IL18, IL12p40, IL23, TLR2, TLR4, and TLR5) did show any significant differences between the patients and the controls in either ileal or duodenal mucosa. However, in JIA, a low mRNA expression of TLR4, TLR2, IL18, TGF-β, FOXP3, and IL10 in the ileum correlated with a high clinical disease activity.

## Conclusions

Based on the analysis of mucosal mRNA expression, decreased anti-inflammatory and immunoregulatory activity in the ileum was associated with a high disease activity of JIA. Occurrence, mechanisms and significance of this potential link between the ileal mucosa and synovial inflammation in JIA should be studied in a more extensive case series including children with JIA but without gastrointestinal symptoms.

